# Green Wraps, Healthy Bites: How Eco-Friendly Packaging Shapes Food Perceived Healthiness and Purchase Intentions

**DOI:** 10.3390/foods15010165

**Published:** 2026-01-03

**Authors:** Chenhan Ruan, Xiaoyang Zhang, Yuanyuan Quan, Tingting Zhang, Xirong Zhao

**Affiliations:** School of Economics and Management, Fujian Agriculture and Forestry University, Fuzhou 350002, China; chenhanruan@126.com (C.R.); z1589673681@163.com (X.Z.); q18779665554@163.com (Y.Q.); q1236892025@126.com (X.Z.)

**Keywords:** eco-friendly packaging, perceived healthiness, utilitarian-hedonic food, environmental consciousness, food marketing

## Abstract

Environmentally friendly packaging has become a widely adopted food retail strategy, yet its impacts on consumer food perceptions (e.g., healthiness, taste, ethical concerns) remain fragmented. Notably, how it shapes healthiness perceptions is understudied. Across three studies, this study demonstrates that consumers often associate eco-friendly packaging with healthiness perception, driven by the heuristic associations of its original-ecology sensory cues, sustainable connotations, and morality signals, which further contribute to purchase intention. In addition, the effect of packaging eco-friendliness on purchase intention is moderated by environmental consciousness and food type. Specifically, the above positive effect of eco-friendly packaging is more prominent for consumers with higher environmental consciousness, and for hedonic (vs. utilitarian) food. The findings advance understanding of how eco-friendly packaging shapes food perceptions, and offer strategic insights for customized packaging design in food marketing.

## 1. Introduction

In today’s digital age, product packaging has become a critical touchpoint in the customer journey. Unlike traditional retail, where shoppers can interact directly with products, online purchases rely heavily on visual packaging information upon delivery [1]. In consideration of consumers’ growing awareness of global environmentalism, eco-friendly packaging has emerged as a widely used marketing strategy, particularly within the food industry. A 2023 report by Trivium reveals that 71% of consumers now actively prioritize products with sustainable packaging, 59% seek explicit information on labels regarding recycling and sustainability, and 80% express interest in refillable packaging products to reduce their environmental impact [2]. Yet, the transition to sustainable packaging is challenging. According to Jason (2022), sustainable packaging may fail to match the required functionality, since some biodegradable materials are perceived as highly moisture-sensitive and may not be safe [3]. Gustavo et al. (2018) note that organizations are still focused on securing economic profits and are uncertain about how such packaging redesigns affect consumer responses [4].

Packaging design, a crucial external attribute of a product, exerts significant influence on food evaluations [5,6]. Given their limited knowledge, consumers may rely on packaging elements such as color, size, material, and shape to form heuristic perceptions [7]. Compared to conventional plastic packaging, eco-friendly packaging refers to packaging solutions that have minimal negative impact on the environment throughout their life cycle, such as bio-based or degradable paper packaging [8]. However, past research has mostly focused on consumers’ overall evaluations of food, yielding conflicting results. Some studies show the positive side of “sustainability bonus” [9,10], but others find neutral or even negative effects due to green-washing skeptics [11,12], inconvenience and functional concerns [13], or a feminine label [14]. This inconsistency partly arises because food evaluation is multi-dimensional (e.g., healthiness, safety, taste, ethical alignment).

Notably, in the present study, we focus on one core attribute of food perceptions, namely, food perceived healthiness. Although little research has confirmed the link between eco-friendliness and healthiness, most studies rely on the halo effect or warm glow effect [15,16] and fail to systematically explore the distinct heuristic association between the two constructs. Differing from the emotionally spillover effect caused by the halo effect or warm glow, heuristic association is a shortcut based on learned connections. We further reveal that the association between eco-friendliness and healthiness is driven by three key attributes of eco-friendly packaging: original-ecology sensory cues, sustainable connotations, and moral brand signals. First, the original-ecology sensory cues are likely to evoke perceptions of naturality, green, and health [17]. Second, the sustainable hints transfer the psychological safety from the environment to individual health [18]. Third, the ethical value of eco-friendly packaging sends out signals of morality, which reinforces consumer trust in health benefits [19]. Moreover, the heuristic association could extend to purchase intention and may vary across different consumer and food characteristics [20,21], so we further propose two moderators for this relationship. First, consumers with stronger environmental consciousness may respond more positively to eco-friendly packaging, amplifying its links to perceived healthiness and purchase intentions. Second, the pathway from eco-friendly packaging to perceived healthiness may be more pronounced for hedonic foods (e.g., indulgent snacks) than utilitarian ones (e.g., groceries), which rely more on subjective perceptions than analytical processing.

Across three studies, we explore how packaging eco-friendliness affects purchase intention via perceived healthiness, and how such effects shift across different contexts. This research makes several contributions. First, prior studies mainly focus on how eco-friendly initiatives affect general food quality and form inconsistent findings [22]. This research identifies the positive effect of eco-friendly packaging on specific dimensions of food perceptions. Second, our research provides a more systematic examination of the heuristic association between eco-friendliness and perceived healthiness, which differs from the predominantly halo effect or warm glow effect. It also systematically identifies the underlying rationale for the heuristic formation and extends such an impact on purchase intention. Third, by examining boundary conditions across individual and food characteristics, it refines the contextual variation conditions for eco-friendly packaging. Finally, we offer actionable insights for businesses to promote eco-friendly packaging and tailor their communication strategies.

## 2. Theoretical Background and Hypotheses Development

### 2.1. The Association Between Packaging Eco-Friendliness and Perceived Healthiness

In the context of food consumption, information asymmetry prevails between consumers and products. Given consumers’ limited knowledge, time, and motivation, they may rely on external cues to infer food quality. Food packaging as a pivotal external cue [23,24], not only conveys fundamental product details but also communicates inherent value through visual symbols, textual materials, and labels [25]. Eco-friendly packaging refers to the package materials and designs that have a minimal negative impact on the environment throughout their lifecycle [26]. Consumers generally perceive paper as more eco-friendly than plastic, and packaging with eco-labels as more sustainable [27,28].

However, previous research has inconsistent findings in terms of consumer responses towards eco-friendly packaging. On the one hand, consumers demonstrate positive attitudes towards eco-friendly packaging. When packaging displays certifications like “organic” or “all-natural”, it brings enhanced health benefits and safety qualities [27,29]. Environmental labels on product packaging can clearly inform consumers about the environmental benefits generated by purchasing the product, making them aware of the significance of their own actions for environmental protection [21]. As a result, consumers prefer paper packaging but harbor negative perceptions toward plastics due to their association that “paper = good, plastic = bad”. On the other hand, some indicate that eco-friendly packaging may reduce purchase intentions due to suspicion and functional concerns. For example, Lynch et al. (2017) mention that bio-based packaging can be partly negatively perceived as green-washing [12]. Macht et al. (2023) note that paper-based food packaging is not always seen as convenient, and consumers do not clearly recognize the advantages of eco-friendly packaging for products like vegetable oil or butter [13].

We propose that these inconsistent findings may stem from the multiple dimensions of food. While prior studies mainly focus on a unidimensional aspect of food quality, food can elicit perceptions such as taste, healthiness, ethics, and convenience, and sometimes consumers may perceive it as favorable in some aspects but unfavorable in others [30]. Notably, we focus on food healthiness as an important attribute. Perceived healthiness is defined as a comprehensive evaluation of a food’s health-related attributes, derived from consumers’ subjective cognition [31]. Moreover, although some studies have revealed the positive impact of sustainable labels on food healthiness perceptions, their underlying mechanisms mainly stem from the warm glow or halo effect [15,16]. These studies fail to systematically explore the cognitive decision shortcut through heuristic information processing perspectives. One exception is Eichin et al. (2025), who examined this heuristic association but did not investigate its effect on purchase intention, nor did they discuss the underlying mechanism and relevant moderators of food type in the context of sustainable packaging [32].

We propose that consumers associate eco-friendly packaging with healthiness perception for three reasons. First, sensory cues related to eco-friendly packaging (e.g., packaging color, paper texture) lead consumers to make more positive ingredient inferences. For example, original-ecology sensory cues on eco-friendly packaging can enhance perceptions of naturality, green, and health [17]. Consumers may infer foods with original-ecology cues to be purer, which in turn enhances their willingness to pay for the product [31,33]. Consumers’ concerns about food ingredients are attenuated, which in turn strengthens their health perceptions. Second, eco-friendly food packaging can evoke consumers’ associations with sustainable connotations. The related concepts, such as naturalness, purity, and sustainability, are inherently linked to healthiness [34]. These sustainable connotations encourage consumers to transfer the positive attributes of the packaging to the product itself, leading them to perceive the product as healthier. Second, the eco-friendly nature serves as an important signal of corporate morality: companies that adopt such packaging are usually viewed as environmentally responsible, and this sense of responsibility may extend to a commitment to consumers’ health [19,21].

Moreover, perceived healthiness is positively related to purchase intention. Prior studies suggest that food healthiness affects consumer decision-making mainly through strengthening value congruence and mitigating consumption risks [35,36]. On the one hand, healthy foods serve as carriers of consumer values, contributing to the formation of a positive social image and strengthening social identity [36]. On the other hand, their safe and high-quality attributes effectively alleviate consumers’ concerns about potential food hazards, reduce perceived consumption risks, and thereby enhance consumer confidence. For example, consumers show higher purchase intention for bio-based food packaging alternatives due to perceived eco-friendliness and convenience [13]. Therefore, we propose the following hypotheses:

**H1.**  *Eco-friendly (vs. non-eco-friendly) packaging leads to higher purchase intention.*

**H2.**  *Perceived healthiness mediates the relationship between packaging eco-friendliness and purchase intention.*

### 2.2. The Moderating Role of Environmental Consciousness

Environmental consciousness refers to individuals’ recognition of ecological issues, sense of responsibility for environmental protection, and tendency to translate pro-environmental beliefs into action. The research indicates that the level of consumers’ environmental consciousness affects their purchasing preferences [37,38]. Individuals with strong environmental consciousness tend to regard eco-friendly attributes as a key criterion for evaluating product quality and place greater emphasis on them [39]. They perceive the eco-friendly attributes of food packaging as a reflection and assurance of the product’s intrinsic quality, leading to higher perceptions of food healthiness [40]. Huang and Lu have demonstrated that enhanced perceived healthiness triggers positive affect toward the product, strengthens trust in its safety and nutritional value, and ultimately elevates purchase intention [41]. However, consumers with lower environmental consciousness are less likely to associate environmentally friendly materials with positive inferences, and instead rely more on other factors such as price [42]. As a result, the association between eco-friendly packaging and perceived healthiness is weakened, leading to lower purchase intention for eco-friendly packaging. Accordingly, we propose the following hypothesis:

**H3.**  *Environmental consciousness moderates the effect of packaging eco-friendliness on purchase intention.*

### 2.3. The Moderating Role of Food Type

The Elaboration Likelihood Model (ELM) suggests that consumers process external information and form attitudes through two distinct routes [43]. When the information consumers encounter is highly relevant to the product’s intrinsic attributes (such as its formula or effectiveness), they tend to process the information through the central route. Under this route, individuals draw on their cognitive motivation and ability to engage in deeper thinking and systematic analysis of the relevant information. In contrast, when product information focuses more on external attributes (such as packaging color or shape), consumers are more likely to rely on the peripheral route to form attitudes or judgments. Under this route, individuals are generally unwilling to invest substantial cognitive resources in deeply processing the information; instead, they tend to rely on external cues or a heuristic approach to reach a judgment quickly [43].

We propose that the association between eco-friendly packaging and food healthiness perception is more prominent for hedonic (vs. utilitarian) food. The purchase motivation for utilitarian food is to obtain specific nutritional or functional benefits [44]. Therefore, when consumers purchase functional foods, they tend to focus more on internal information related to health, such as ingredients and functional benefits, read and evaluate nutrient content, and rely more on rational analysis to make decisions [45]. In contrast, for hedonic food, consumers are more driven by the emotional arousal and subjective feelings [44]. This prompts consumers to rely more on extrinsic sensory cues such as appearance, aroma, and packaging cues unrelated to internal attributes of food to make choices. In other words, they are more likely to process information in a heuristic way, and thus the eco-friendly label exerts greater influence on food healthiness perception and purchase intention. Accordingly, we propose the following hypothesis:

**H4.**  *Food type moderates the effect of packaging eco-friendliness on purchase intention.*

**Figure 1:**
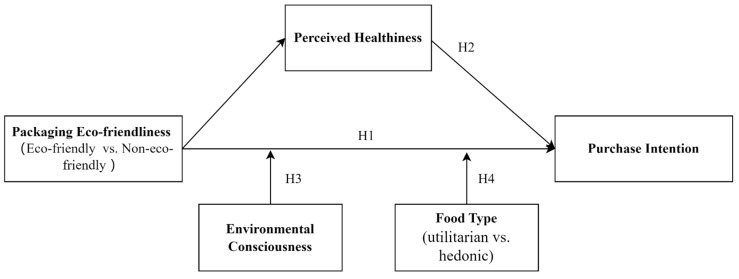
Research framework.

Overall, our research model is shown in Figure 1.

## 3. Study Overview

We adopted the experimental method commonly used in consumer behavior research to test the above hypotheses. The experimental method is widely used to examine causal relationships between variables, as it can create a scenario to control for the effect of confounding variables. In order to increase the external validity, we used different types of food stimuli and recruited different participants across studies. The participants took part in only one of the three experiments. To ensure that the sample sizes of the three studies were sufficient, we used G*Power 3.1 to determine the minimum required sample size [46]. In addition, we analyzed the experimental results using the Statistical Package for the Social Sciences (SPSS), which is widely used for data processing in consumer research. The detailed information for Studies 1–3 is presented in Table 1 (see Table 1). All participants signed informed consent, and all studies were approved by the Institutional Review Board of Fujian Agriculture and Forestry University (detailed information can be found at the end of the manuscript).

## 4. Study 1: Main Effect and Mediating Effect

The purpose of Study 1 is to test the main effect and underlying mechanism of perceived healthiness. We hypothesized in H1 and H2 that consumers are more likely to purchase products with eco-friendly packaging due to perceived healthiness. In addition, we ruled out confounding variables such as naturalness, perceived fit, and perceived firm ethicality.

### 4.1. Methodology

#### 4.1.1. Participants

Study 1 employs a factorial between-subjects design with two types of packaging (eco-friendly vs. non-eco-friendly). Given that no prior study has examined the effect of eco-friendly packaging on consumers’ purchase intentions, a medium effect size (Cohen’s f = 0.25) with statistical power (1-β) greater than 0.80 was used to determine the sample size. A minimum sample size of 64 participants in each group (128 in total) was determined using the G*Power 3 program. We recruited 200 participants through the Credamo platform (www.credamo.com). Among the 200 participants (54.5% female, M_age_ = 30.23, SD_age_= 7.76), 43.5% held a bachelor’s degree.

#### 4.1.2. Materials and Procedures

All participants provided informed consent and were randomly assigned to either the eco-friendly or non-eco-friendly condition (N_eco-friendly_ = 100, N_non-eco-friendly_ = 100). All participants were instructed to imagine that they were browsing juicy drinks from a company on an e-commerce platform. Then, participants were presented with an image of the product along with a brief description to deliver the manipulation. The two groups viewed the same product, but the packaging material and product information varied. In terms of packaging materials, we selected two types: PET plastic and biodegradable paper. Polyethylene Terephthalate (PET) is a high-performance thermoplastic that inherently possesses excellent barrier properties against gases, heat, and acids. However, as it is primarily derived from fossil fuels and remains non-biodegradable in the natural environment, it is not environmentally friendly [47]. In contrast, paper packaging provides clear environmental benefits because it uses biobased and biodegradable coatings, which help reduce the environmental burden [48]. Moreover, in the eco-friendly condition, the juicy drink is contained in a biodegradable paper bottle with a recycling symbol. However, in the non-eco-friendly group, the bottle was made of PET plastic materials without a recycling symbol (see Appendix A).

After that, all participants reported their purchase intention, perceived healthiness, naturalness, perceived fit, and perceived firm ethicality. All the aforementioned variables were measured using mature scales. Specifically, purchase intention was measured by asking participants: “The probability I would consider buying this product is high”; “I would buy this product”; “It is acceptable for me to pay more money for this product” (1 = not at all; 7 = very much; Cronbach’s alpha = 0.86; adapted from Chang & Chen, 2008) [49]. Perceived healthiness was measured through five items: “To what extent do you think the food is healthy/nutritious/good for body/fatty (reverse coded)/high in calories (reverse coded)” (1 = not at all; 7 = very much; Cronbach’s alpha = 0.90; adapted from Hagen, 2020) [34]. Naturalness was measured by asking participants to rate the degree of the product’s perceived naturalness (1 = very low; 7 = very high; adapted from Pichierri & Pino, 2023) [50]. Perceived fit was measured through four items: “The packaging material is suitable for the characteristics of this juice”; “The packaging is compatible with the quality of the product”; “The packaging material fits your expectations of the juice”; “The packaging material is well-matched to the product” (1 = strongly disagree; 7 = strongly agree; Cronbach’s alpha = 0.95; adapted from Lee & Kim, 2024) [51]. Perceived firm ethicality was measured through three items: “The enterprise follows ethical norms”; “The enterprise handles problems in ethical ways”; “You recognize the ethical behaviors of the enterprise” (1 = completely disagree; 7 = completely agree; Cronbach’s alpha = 0.88; adapted from Wimbush et al., 1997) [52]. Then, participants were asked to complete the manipulation check of the perceived eco-friendliness with two items: “To what extent do you think the packaging of this product is eco-friendly” and “To what extent do you think the package can be recycled” (1 = not at all; 7 = very much; Cronbach’s alpha = 0.93; adapted from Ollitervo et al., 2025) [53]. Lastly, participants provided their demographic information, such as age, gender, and education.

### 4.2. Results

#### 4.2.1. Manipulation Check

We conducted an independent-samples *t*-test using SPSS 26, a commonly used method for examining whether the means of two independent groups differ significantly. The independent *t*-test indicates that participants in the eco-friendly group perceive stronger eco-friendliness compared to those in the non-eco-friendly group (M_eco-friendly_ = 5.36, SD_eco-friendly_ = 1.92; M_non-eco-friendly_ = 2.60, SD_non-eco-friendly_ = 1.81; t (198) = 10.48, *p* < 0.001). Thus, the manipulation is successful.

#### 4.2.2. Main Effect

We also used an independent-samples *t*-test to examine differences in purchase intention between participants in the eco-friendly and non-eco-friendly packaging groups. The results indicate that participants in the eco-friendly group demonstrate higher purchase intention (M_eco-friendly_ = 5.38, SD_eco-friendly_ = 1.03) than those in the non-eco-friendly group (M_non-eco-friendly_ = 3.64, SD_non-eco-friendly_ = 0.99; t (198) = 12.09, *p* < 0.001). Thus, H1 is supported.

#### 4.2.3. Mediating Effect

First, we conducted an independent-samples *t*-test. The results show a significant difference in perceived healthiness between the two groups (M_eco-friendly_ = 5.53, SD_eco-friendly_ = 0.99; M_non-eco-friendly_ = 3.99, SD_non-eco-friendly_ = 1.12, t (198) = 10.35, *p* < 0.001). Then, we employed the bootstrapping method in PROCESS (Model 4; Hayes 2017) to test the mediating role of perceived healthiness in the relationship between packaging eco-friendliness and purchase intention [54]. Specifically, packaging eco-friendliness (0 = eco-friendly, 1 = non-eco-friendly) was set as the independent variable, purchase intention as the dependent variable, and perceived healthiness as the mediator. The analysis was conducted with 5000 bootstrap samples and a 95% confidence interval. The results indicated that there is a significant mediating effect of perceived healthiness in the relationship between packaging eco-friendliness and purchase intention (Indirect Effect = 0.54, Standard Error = 0.13, 95% CI = [0.2799, 0.8143]). The confidence interval did not include zero (LLCI = 0.2799, ULCI = 0.8143), indicating a significant indirect effect of perceived healthiness. Subsequently, we added naturalness, perceived fit, and perceived firm ethicality as control variables, and after controlling for that, the result remains unchanged (Indirect Effect = 0.10, Standard Error = 0.05; 95% CI = [0.0048, 0.2148]). The confidence interval did not include zero (LLCI = 0.0048, ULCI = 0.2148), indicating the indirect effect of perceived healthiness was still significant after ruling out the alternative explanations. Thus, H2 is supported.

## 5. Study 2: The Moderating Effect of Environmental Consciousness

Study 2 employs a two-factorial (packaging eco-friendliness: eco-friendly vs. non-eco-friendly) between-subjects design. This study aims to replicate the findings of Study 1 and validate the moderating effect of environmental consciousness. Specifically, we proposed that the eco-friendly food packaging leads to greater healthiness perception and purchase intention among all participants; however, for individuals with higher environmental consciousness, such a positive effect is more elaborated. In addition, we ruled out the confounding effects of packaging attractiveness, consumers’ general preference for healthy food, and taste perception.

### 5.1. Methodology

#### 5.1.1. Participants

We recruited 200 participants from Credamo, excluded 23 invalid samples that failed the attention check, and finally obtained a sample of 177 participants (61.6% female, M_age_ = 31.10, SD_age_ = 9.02); 70.1% had a bachelor’s degree. All participants signed the informed consent online and received a small monetary compensation after completion. All participants in this study were newly recruited and had not taken part in Study 1 before. Study 2 was approved by the Institutional Review Board (IRB) of Fujian Agriculture and Forestry University.

#### 5.1.2. Materials and Procedures

Participants were told that they were going to purchase a yogurt online. They were randomly assigned to one of two groups (N_eco-friendly_ = 89, N_non-eco-friendly_ = 88) and viewed an introduction to a yogurt along with its packaging. We manipulated packaging eco-friendliness by adding a recycling symbol to the yogurt container. Specifically, the yogurt packaging shown to participants in the eco-friendly condition displayed a recycling symbol, whereas the packaging shown to those in the non-eco-friendly condition did not (see Appendix B).

Then, we measured participants’ purchase intention, perceived healthiness, taste perception, and packaging attractiveness. All the aforementioned variables were measured using mature scales. For example, purchase intention was measured through three items: “To what extent do you think you will buy this product”; “To what extent do you want to buy this product”; “Overall, to what extent are you willing to buy this product” (1 = not at all; 7 = very much; Cronbach’s alpha = 0.90; adapted from Chang & Chen, 2008) [49]. Perceived healthiness was measured through three items: “To what extent do you think the food is healthy/nutritious/good for body” (1 = not at all; 7 = very much; Cronbach’s alpha = 0.86; adapted from Hagen, 2020) [34]. Taste perception was measured, such as “To what extent do you think this yogurt is tasty” (1 = not at all; 7 = very much; adapted from Hallez et al., 2023) [55]. Packaging attractiveness was measured, such as “To what extent do you think the packaging design is attractive” (1 = not at all; 7 = very much; adapted from Wang et al., 2024) [24].

After that, participants completed the manipulation check of the perceived packaging eco-friendliness: “To what extent do you think this packaging is friendly to the environment”; “To what extent do you think the manufacturing and disposal of this packaging causes less harm to the environment”; “To what extent do you think this packaging is relatively more eco-friendly than other packaging”; and “To what extent do you think this packaging deserves to be labeled environmentally friendly” (1 = not at all; 7 = very much; Cronbach’s alpha = 0.97; adapted from Sokolova et al., 2023) [28]. Next, we measured participants’ environmental consciousness by asking them to what extent they agree that, “I believe that individual behavior is crucial for environmental protection”; “For the sake of environmental protection, society should prioritize ecological benefits over economic benefits”; and “Human over-exploitation of natural resources will lead to irreversible environmental disasters” (1 = not at all; 7 = very much; Cronbach’s alpha = 0.60; Schlegelmilch et al., 1996) [56]. We measured consumers’ general preference for healthy foods by asking them how much they prefer unhealthy foods (1 = not at all; 7 = very much). Finally, all participants reported their demographic information, including gender, age, and educational background.

### 5.2. Results

#### 5.2.1. Manipulation Check

The results of the independent samples *t*-test indicate a significant difference between the two groups in perceptive eco-friendliness (M_eco-friendly_ = 6.08, SD_eco-friendly_ = 0.76; M_non-eco-friendly_ = 2.61, SD_non-eco-friendly_ = 1.66, t (175) = 17.93, *p* < 0.001), which confirms a successful manipulation.

#### 5.2.2. Mediating Effect

The results of the independent samples *t*-test show a significant difference in perceived healthiness between the two groups (M_eco-friendly_ = 5.79, SD_eco-friendly_ = 0.71; M_non-eco-friendly_ = 4.98 SD_non-eco-friendly_ = 1.21, t (175) = 5.45, *p* < 0.001). Then, we used the Process model to test the mediating effect of perceived healthiness (5000 samples, Model 4, 95% confidence interval; Hayes, 2017) [54]. Specifically, we set packaging eco-friendliness (0 = eco-friendly, 1 = non-eco-friendly) as the independent variable, purchase intention as the dependent variable, and perceived healthiness as the mediator. The results indicated that there is a significant indirect effect of perceived healthiness (Indirect effect = 0.40, Standard Error = 0.11, 95% CI = [0.2002, 0.6487]). The confidence interval did not include zero (LLCI = 0.2002, ULCI = 0.6487), indicating a significant mediating effect of perceived healthiness. After that, we set taste perception, packaging attractiveness, and general preference for healthy food as control variables, and the effect remains unchanged (Indirect effect = 0.11, Standard Error = 0.06, 95% CI = [0.0054, 0.2252]). The confidence interval did not include zero (LLCI = 0.0054, ULCI = 0.2252), indicating the mediating effect of perceived healthiness was significant after ruling out potential alternative explanations. Thus, H2 is supported.

#### 5.2.3. Moderating Effect

**Figure 2:**
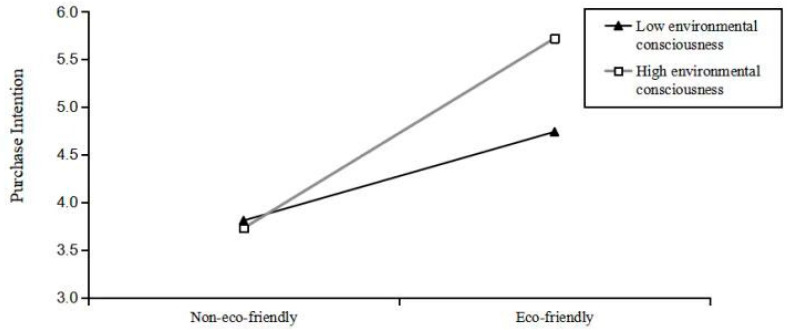
The moderating effect of environmental consciousness.

We conducted a moderating effect analysis using SPSS. 26 Model 1 of the PROCESS macro (Hayes, 2017) [54], with a bootstrapping sample size of 5000 to validate the moderating role of environmental consciousness. Specifically, we set the packaging type (0 = non-eco-friendly; 1 = eco-friendly) as the independent variable, consumers’ environmental consciousness as the moderator, and purchase intention as the dependent variable. The results show that consumers’ environmental consciousness significantly moderates the relationship between packaging eco-friendliness and purchase intention (Effect = 0.72, Standard Error = 0.28, 95% CI = [0.1720, 1.2743], *p* = 0.010). The confidence interval did not include zero (LLCI = 0.1720, ULCI = 1.2743), indicating that the moderating effect of environmental consciousness is significant. Additionally, we conducted post hoc comparisons to further examine differences in participants’ purchase intentions for products with different packaging under conditions of high versus low environmental consciousness. Specifically, among consumers with higher environmental consciousness, eco-friendly packaging significantly increases purchase intention (Effect = 1.99, Standard Error = 0.29, t = 6.94, *p* < 0.001, 95% CI = ([1.4271, 2.5626]). However, for consumers with lower environmental consciousness, the positive effect of eco-friendly packaging is attenuated (Effect = 0.93, Standard Error = 0.29, t = 3.22, *p* = 0.015, 95% CI = [0.3610, 1.5035]). Therefore, H3 is confirmed (see Figure 2).

## 6. Study 3: The Moderating Effect of Food Type

The purpose of Study 3 is to replicate the findings of the prior studies and explore the moderating effect of food type (utilitarian vs. hedonic). In addition, we ruled out the potential confounding effect of packaging complexity and packaging typicality.

### 6.1. Methodology

#### 6.1.1. Participants

The sample size was determined based on an F-test (analysis of covariance with fixed effects, main effects, and interactions), considering a medium effect size of 0.25, and a statistical power of 0.80. The minimum sample size was 45 participants (180 in total). We recruited 200 participants from Credamo (https://www.credamo.com), who provided their informed consent and received a small monetary compensation for participation. After excluding 17 invalid questionnaires due to failure in the attention check, the remaining 183 valid responses were randomly assigned to one of four experimental conditions. Among the 183 valid participants (65.6% female, M_age_ = 31.89, SD_age_ = 9.20), 61.7% of them had a bachelor’s degree. The participants in this study were entirely new and did not participate in the previous two studies. Study 3 was approved by the Institutional Review Board (IRB) of Fujian Agriculture and Forestry University.

#### 6.1.2. Materials and Procedures

Participants were randomly assigned to one of four groups in a 2 (packaging eco-friendliness: eco-friendly vs. non-eco-friendly) × 2 (food type: utilitarian vs. hedonic) between-subjects design. Participants were asked to imagine that they intend to purchase food in an e-commerce platform (M_eco-friendly utilitarian_ = 45, M_eco-friendly hedonic_ = 44, M_non-eco-friendly utilitarian_ = 47, M_non-eco-friendly hedonic_ = 47), and then they saw the packaging of the food similar to that in Study 1 to deliver the manipulation of eco-friendliness (see Appendix C). We manipulated packaging eco-friendliness by varying the packaging materials of the food products. Specifically, participants in the eco-friendly packaging condition viewed products packaged in more sustainable, biodegradable paper materials that have a lower negative impact on the environment. In contrast, participants in the non-eco-friendly packaging condition viewed products packaged in PET plastic, which is relatively less environmentally friendly [47,48]. For food type manipulation, we used dried okra and chocolate as the experimental materials for utilitarian and hedonic food.

Then, participants reported purchase intention (Cronbach’s alpha = 0.86; adapted from Chang & Chen, 2008) [49] and perceived healthiness (Cronbach’s alpha = 0.90; adapted from Hagen, 2020) [34] consistent with Study 1. These variables were all adapted from mature scales. Then, packaging complexity was measured by asking them, “To what extent do you think the packaging design is visually complex” (adapted from Wang et al., 2024) [24]. Packaging typicality is measured by asking them: “To what extent do you think the packaging design is typical” (adapted from Marozzo et al., 2020) [57]. After that, we delivered the manipulation check of packaging eco-friendliness similar to prior studies. We also asked participants: “To what extent do you think the food is more utilitarian or hedonic?” (1 = completely utilitarian; 7 = completely hedonic; adapted from Kivetz & Zheng, 2017) to deliver the manipulation check for food type [58]. Finally, all participants reported their demographic information, including gender, age, and educational background.

### 6.2. Result

#### 6.2.1. Manipulation Checks

The results of the independent samples *t*-test indicate that participants in the eco-friendly group (M_co-friendly_ = 5.84, SD_eco-friendly_ = 1.10) perceived the packaging as more eco-friendly than those in the non-eco-friendly group (M_non-eco-friendly_ = 3.44, SD_non-eco-friendly_ = 2.07, t (181) = 9.76, *p* < 0.001). Participants in the hedonic food group perceived the food as having higher hedonic attributes than those in the utilitarian food group (M_hedonic_ = 5.78, SD_hedonic_ = 1.32; M_utilitarian_ = 2.32, SD_utilitarian_ = 1.44; t (181) = 16.97, *p* < 0.001). Thus, the manipulations are successful.

#### 6.2.2. Mediating Effect

The result of the independent samples *t*-test shows a significant difference in perceived healthiness between the two groups (M_eco-friendly_ = 4.63, SD_eco-friendly_ = 1.34; M_non-eco-friendly_ = 4.12, SD_non-eco-friendly_ = 1.54, t (181) = 2.39, *p* = 0.018). Then, a Bootstrapping Process model 4 by Hayes (2017) verifies the mediating effect of perceived healthiness [54]. Specifically, packaging eco-friendliness (0 = eco-friendly, 1 = non-eco-friendly) was set as the independent variable, purchase intention as the dependent variable, and perceived healthiness as the mediator. The analysis was conducted with 5000 bootstrap samples and a 95% confidence interval. The results indicated that there is a significant mediating effect of perceived healthiness (Indirect effect = 0.10, Standard Error = 0.06, 95% CI = [0.0101, 0.2338]). The confidence interval did not include zero (LLCI = 0.0101, ULCI = 0.2338), indicating the mediating effect of perceived healthiness was significant. After controlling for variables such as packaging complexity and packaging typicalness, the mediating effect of perceived healthiness remains unchanged (Indirect effect = 0.14, Standard Error = 0.06, 95% CI = [0.0288, 0.2753]). The confidence interval does not include zero (LLCI = 0.0288, ULCI = 0.2753), indicating that perceived healthiness mediates the relationship between packaging eco-friendliness and purchase intention, while ruling out alternative explanations such as packaging complexity and packaging typicality. Thus, H2 is supported.

#### 6.2.3. Moderating Effect

**Figure 3:**
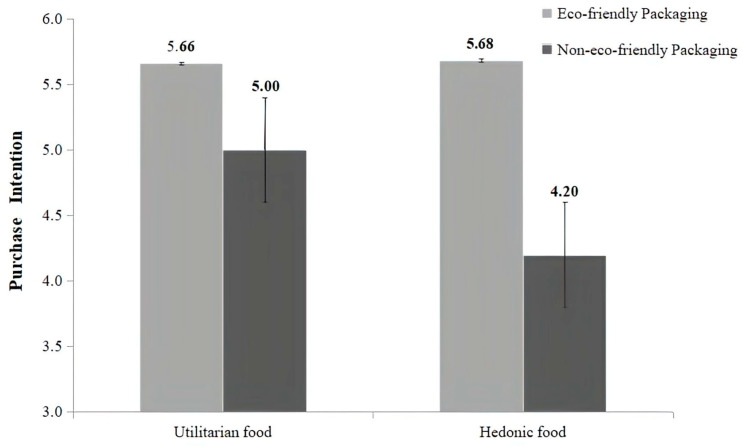
Moderating effect of food type on purchase intention.

Study 3 uses a two-way ANOVA to analyze the interaction effect between packaging eco-friendliness and food type on purchase intention. The results indicate a significant interaction effect (F (1, 179) = 6.19, *p* = 0.014, η^2^ = 0.033). The post hoc comparison indicates that for hedonic food, there is a significant difference in purchase intention between the eco-friendly and the non-eco-friendly group (M_eco-friendly_ = 5.68, SD_eco-friendly_ = 0.66; M_non-eco-friendly_ = 4.20, SD_non-eco-friendly_ = 1.50, *p* < 0.001). However, for functional foods, the difference in purchase intention between the two packaging types becomes less pronounced (M_eco-friendly_ = 5.65, SD_eco-friendly_ = 0.76; M_non-eco-friendly_ = 5.00, SD_non-eco-friendly_ = 1.29, *p* = 0.005), and this provides preliminary support for H4 (see Figure 3).

To further verify the moderating effect of food type, we employed the bootstrapping method in PROCESS (Model 1; Hayes, 2017) [54]. Specifically, packaging eco-friendliness was set as the independent variable, purchase intention as the dependent variable, and food type as the moderator. The analysis was conducted with 5000 bootstrap samples and a 95% confidence interval. The results showed that food type significantly moderates the effect of packaging eco-friendliness on purchase intention (Moderating Effect = 0.82, Standard Error = 0.33, *p* = 0.014, 95% CI = [0.1706, 1.4773]). The confidence interval does not include zero (LLCI = 0.1706, ULCI = 1.4773), indicating that the moderating effect of food type is significant.

Moreover, we conducted a moderated mediation analysis using the PROCESS Model 8 (Hayes, 2017) [54], with a bootstrap sample of 5000, in which packaging eco-friendliness (0 = non-eco-friendly; 1 = eco-friendly) as the independent variable, food type (0 = utilitarian food; 1 = hedonic food) as the moderator, perceived healthiness was the mediator, and purchase intention was the dependent variable. The results confirm a significant moderated mediation effect (Effect = 0.14, Standard Error = 0.08, 95% CI: [0.0063, 0.3311]). In particular, there is a significant mediation effect of perceived healthiness in the hedonic food condition (Effect = 0.19, Standard Error = 0.09, 95% CI: [0.0451, 0.3965). But in the utilitarian food condition, the effect is no longer significant (Effect = 0.06, Standard Error = 0.04, 95% CI: [−0.0100, 0.1563]). Thus, H4 is supported.

## 7. Discussion

### 7.1. Theoretical Contributions

This research makes three distinct theoretical contributions. First, past research on the effects of eco-friendly packaging on food evaluation has yielded inconsistent findings. Some studies suggest that eco-friendly packaging can enhance consumers’ moral perceptions and thereby lead to more favorable evaluations [16]. Whereas other research finds that eco-friendly packaging may trigger green-washing concerns [11,12], resulting in opposite conclusions. This study finds that eco-friendly packaging positively impacts one key dimension of food perception (i.e., perceived healthiness).

Secondly, our research provides a more systematic examination of the heuristic association between eco-friendliness and perceived healthiness. Although some studies documented such an association, they primarily rely on emotional spillover from warm glow or halo effect [15,16]. In contrast, our study adopts a cognitive perspective showing the heuristic association between the two constructs. This also extends Eichin et al. (2025)’s work by examining the heuristic approach [32]. But further, we clarify the underlying mechanisms of heuristic association, extend the effect to purchase intention, and examine the influential factors such as product type. By doing so, we offer a more comprehensive understanding of consumers’ shortcuts in eco-friendly packaging, along with their driving mechanisms, influential factors, and impacts on behavioral indicators.

Additionally, our research examines how the belief that “eco-friendly equals healthy” varies across different consumers and product types. On the one hand, although prior research has examined the moderating role of environmental consciousness, it has been found to be a weak and sometimes insignificant moderator [15,32]. In contrast, our study finds that in the context of eco-friendly packaging, consumers exhibit higher trust in the eco-friendly cues presented on the packaging, resulting in a generally stronger moderating effect. On the other hand, existing research suggests that consumers’ purchase intentions for eco-friendly packaging are high for healthy products, partly due to increased perceptions of satiety [59]. However, we find that eco-friendly packaging has a stronger effect on hedonic food than on utilitarian food. This is because consumers are more likely to rely on a heuristic approach when making decisions for hedonic products.

### 7.2. Managerial Implications

The findings of this research offer valuable insights for businesses in package design, especially in e-commerce platforms. First, firms should consider the advantages of eco-friendly packaging. On the one hand, brands can highlight the environmental attributes of packaging through explicit display of authoritative eco-certifications alongside simple explanations (e.g., naturally degrades in 3 months) to enhance the perceived environmental friendliness of food packaging. On the other hand, firms can leverage both tactile and visual cues to enhance authenticity and reduce uncertainty. For example, they can employ some sensory cues, such as adopting transparent packaging (i.e., see-through containers) that allow consumers to directly observe the product.

Second, brands can proactively tailor the communication strategies of eco-friendly products to consumers’ environmental consciousness in food marketing. For consumers with high environmental consciousness, emphasizing eco-friendly packaging can directly drive purchase intent. Messages such as “Packaged in plant-based materials, because your health and the planet matter” not only reinforce the brand’s commitment to sustainability but also strengthen positive associations with the product’s health benefits. However, for consumers with low environmental consciousness, brands can highlight the link between eco-friendly packaging and food healthiness through targeted reinforcement measures. For example, product advertisements could include content emphasizing the connection between eco-friendly packaging and nature, thereby strengthening the perceived association between packaging and food healthiness. In addition, brands may need to consider other practical benefits (e.g., convenience, freshness) while gradually conveying the value of eco-friendly packaging to consumers.

Finally, firms should align packaging strategies with product type (utilitarian vs. hedonic) to address consumer priorities. For utilitarian products (e.g., probiotics yogurt, whole grains), consumers pay attention to utilitarian attributes, so brands should pair eco-friendly packaging with clear, prominent health labels (e.g., organic, low-sugar, nutritional content) to improve purchase intention. For hedonic products (e.g., indulgent snacks or desserts), consumers tend to prefer tasty options, with the perception that such tastiness is linked to unhealthiness. Eco-friendly packaging can act as a critical compensating cue to mitigate consumers’ concerns about the product’s healthiness. This strategy helps to ensure that their hedonic choice does not come at the cost of healthiness perceptions.

### 7.3. Limitations and Future Research

While this research provides valuable insights into how eco-friendly packaging influences consumer perceptions and purchase intentions for foods, several limitations open avenues for future explorations. First, although we adopt mature scales in experimental designs, consumer perceptions are mainly based on self-reported measures, and they do not capture actual purchase behavior in real settings. Future research could extend our findings in the real world with more objective data, such as real purchases, consumer click-through rates, or eye-tracking movements towards advertising elements on e-commerce platforms. In addition, various types of eco-friendly packaging exist in real-life settings, such as bio-based materials and biodegradable paper. Consumers may also perceive these types of eco-friendly packaging differently. Future research could therefore further examine how different forms of eco-friendly packaging influence consumer purchase behavior.

Second, our study focuses on healthiness as the key dimension of food perception affected by eco-friendly packaging. However, food quality perception is inherently multi-dimensional, encompassing aspects such as taste expectations, ethical value, and perceived quality [30], which could interact with eco-friendly packaging in distinct ways. Although we rule out perceptions on taste and naturalness, future research could investigate how eco-friendly packaging impacts diverse food perceptions, such as ethical value, convenience, freshness, and taste concerns, to provide a more comprehensive understanding of its role in shaping consumer evaluations.

Moreover, future studies could further delve into the contextual variations that shape consumers’ preferences for eco-friendly packaging. While our current study has examined key factors such as environmental consciousness and product type (utilitarian vs. hedonic), other types of individual and food characteristics could serve as meaningful boundaries. For example, price sensitivity may reduce preferences for eco-friendly packaging [60]. Additionally, for perishable foods, packaging’s primary purpose is to preserve freshness and ensure safety [61]. Consumers may prioritize these utilitarian needs over eco-friendliness, which may be less effective at blocking bacteria.

## 8. Conclusions

Across three studies, our results suggest that eco-friendly packaging can enhance purchase intention via perceived healthiness, as the eco-friendly packaging design delivers original-ecology sensory cues, sustainable connotations, and morality signals. In Study 1, we establish the main effect and uncover the underlying mechanism, demonstrating that consumers tend to associate eco-friendly packaging with food healthiness perception. Moreover, the effectiveness of eco-friendly packaging differs across various contextual factors. Study 2 identifies consumers’ environmental consciousness as a key moderator. Although consumers generally prefer foods with eco-friendly packaging, the preference is more elaborate for consumers with higher environmental consciousness. Study 3 further demonstrates that compared to utilitarian food, the positive effect of eco-friendly packaging on purchase intention is more pronounced for hedonic food. This is because consumers are more likely to use heuristic information processing rather than an analytical approach when making hedonic food decisions.

## Figures and Tables

**Table 1:** Study overview.

Study	Product	Purpose	Measures	Results
Study 1 (N = 200)	Juicy drinks	Testing the main effect of packaging eco-friendliness on purchase intention (H1), and the mediating effect of perceived healthiness (H2).	Purchase intention. Perceived healthiness.	Purchase intention: M_eco-friendly_ = 5.38, SD_eco-friendly_ = 1.03, M_non-eco-friendly_ = 3.64, SD_non-eco-friendly_ = 0.99, t (198) = 12.09, *p* < 0.001. Perceived healthiness: M_eco-friendly_ = 5.53, SD_eco-friendly_ = 0.99, M_non-eco-friendly_ = 3.99, SD_non-eco-friendly_ = 1.12, t (198) = 10.35, *p* < 0.001
Study 2 (N = 177)	Yogurt	Testing the main effect (H1), the mediating effect (H2), and the moderating effect of environmental consciousness between packaging eco-friendliness and purchase intention (H3).	Purchase intention. Perceived healthiness. Environmental consciousness.	Purchase intention: M_eco-friendly_ = 5.25, SD_eco-friendly_ = 1.52, M_non-eco-friendly_ = 3.77, SD_non-eco-friendly_ = 1.22, t (175) = 7.38, *p* < 0.001. Perceived healthiness: M_eco-friendly_ = 5.79, SD_eco-friendly_ = 0.71, M_non-eco-friendly_ = 4.98, SD_non-eco-friendly_ = 1.21, t (175) = 5.45, *p* < 0.001. Moderating effect of environmental consciousness: Effect = 0.72, Standard Error = 0.28, *p* = 0.010, 95% CI = [0.1720, 1.2743].
Study 3 (N = 183)	Dried okra and chocolate	Testing the main effect (H1), the mediating effect (H2), and the moderating effect of food type (utilitarian vs. hedonic) between packaging eco-friendliness on purchase intention (H4).	Purchase intention. Perceived healthiness.	Purchase intention: M_eco-friendly_= 5.67, SD_eco-friendly_ = 0.71, M _non-eco-friendly_= 4.60, SD_non-eco-friendly_ = 1.45; t (181) = 6.30, *p* <0.001. Perceived healthiness: M_eco-friendly_ = 4.63, SD_eco-friendly_ = 1.34, M_non-eco-friendly_ = 4.12, SD_non-eco-friendly_ = 1.54; t (181) = 2.39, *p* = 0.018. Moderating effect of food type: Effect = 0.82, Standard Error = 0.33, *p* = 0.014, 95% CI = [0.1706, 1.4773].

**Figure A1:**
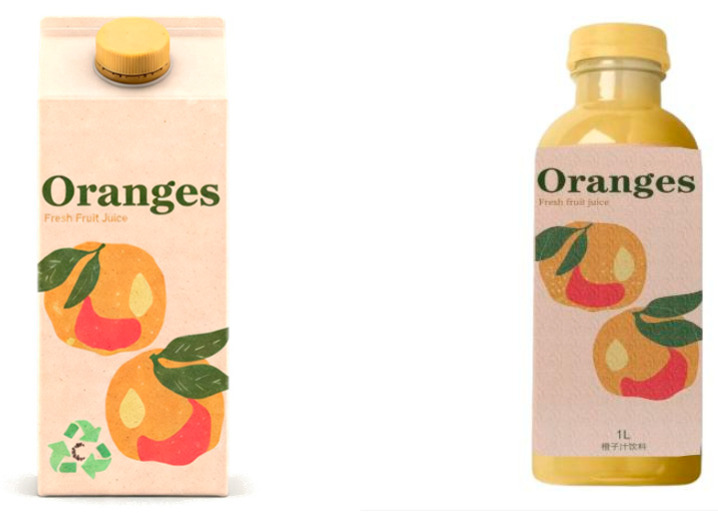
The image on the left depicts eco-friendly packaging, whereas the image on the right illustrates non-eco-friendly packaging. Packaging eco-friendliness was manipulated by varying the material of the juice containers and the presence of a recycling symbol. Specifically, the eco-friendly packaging is made from a more sustainable paper material and displays a recycling symbol. In contrast, the non-eco-friendly packaging is made from low-sustainability PET plastic and does not feature a recycling symbol.

**Figure A2:**
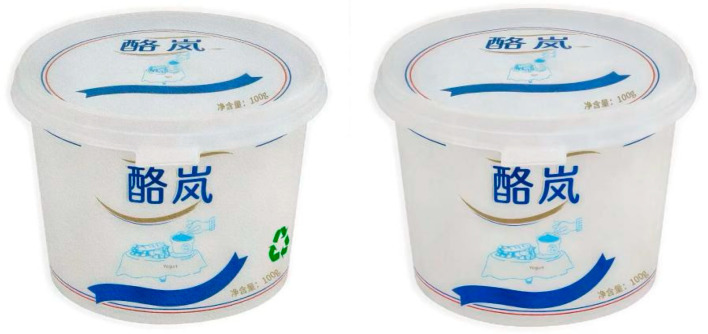
The image on the left depicts eco-friendly packaging, whereas the image on the right illustrates non-eco-friendly packaging. Packaging eco-friendliness was manipulated by using a recycling symbol on the yogurt container. Specifically, the eco-friendly packaging displays a recycling symbol printed on the yogurt container, while the non-eco-friendly packaging does not.

## Data Availability

The original contributions presented in this study are included in the article. Further inquiries can be directed to the corresponding author.

## Appendix C. Manipulation Stimulus in Study 3

**Food Type**	**Hedonic Food**	**Stimuli**	**Packaging TYPE**	
			**Eco-Friendly Packaging**	**Non-Eco-Friendly** **Packaging**
		**“VERSUES” is a chocolate brand. Its products feature rich aroma and smooth flavor, delivering sweet enjoyment and emotional satisfaction for a pleasurable, indulgent experience.**	****	****
	**Utilitarian Food**	**“VERSUES” is a dried okra brand. Its products feature nutritional value and health benefits, delivering satiety and energy support for sustained physical well-being.**	****	****
		***Note*:** We manipulated packaging eco-friendliness by varying the packaging materials of the foods. The eco-friendly packaging is made from more sustainable, biodegradable paper materials that have a lower environmental impact. In contrast, the non–eco-friendly packaging is made from low-sustainability PET plastic materials and is therefore less environmentally friendly.		
